# New species of *Furculanurida* (Collembola, Neanuridae, Pseudachorutinae) from the Luquillo Mountains, Puerto Rico

**DOI:** 10.3897/zookeys.917.33020

**Published:** 2020-03-09

**Authors:** Claudia M. Ospina-Sánchez, José G. Palacios-Vargas, Grizelle González

**Affiliations:** 1 USDA-FS, International Institute of Tropical Forestry, Río Piedras, PR 00926-1119, México United States Department of Agriculture - Forest Service Río Piedras United States of America; 2 Laboratorio de Ecología y Sistemática de Microartrópodos, Departamento de Ecología y Recursos Naturales, Facultad de Ciencias, Universidad Nacional Autónoma de México, 04510 México, D.F., México Universidad Nacional Autónoma de México México Mexico

**Keywords:** Island, Luquillo Experimental Forest, subtropical forest, taxonomy

## Abstract

A new species of *Furculanurida* is described and illustrated. *Furculanuridabistribus***sp. nov.** differs from other species of the genus by the presence of three eyes, three setae on the dens, and the white and purple coloration pattern. A key for identification of the world species of the genus is included.

## Introduction

In Puerto Rico, most studies of arthropod community dynamics have been done in the Luquillo Mountains. Designated as a US Experimental Forest in 1956, it became part of the International Network of Biosphere Reserves in 1976 (González and Barberena 2018; Quiñones et al. 2018; Richardson 1999). Four easily distinguishable forest types are dominated by an assortment of distinctive tree species. The Tabonuco forest (*Dacryodesexcelsa* Vahl) occupies areas below 600 m, in the mid-elevation zone. Palo Colorado forest (*Cyrillaracemiflora* L.) occurs in areas above the cloud condensation level from 600 to 900 m a.s.l. The Elfin forest (dominant tree *Tabebuiarigida* Urban), with stunted vegetation and waterlogged anoxic soils, is located only on the highest peaks above 900 m. Palm forests (*Prestoeamontana* (R. Grah.) Nichols) occur at all elevations, predominantly on windward slopes, in wet gullies, and in stream valleys (Gould et al. 2006). These forests represent subtropical wet and subtropical rain forest life zones in Puerto Rico (Ewel and Whitmore 1973).

In most studies of litter and soil fauna in the Luquillo Experimental Forests (hereafter LEF), Collembola are an important group because of their numerical dominance, combined with their key responses to changes in disturbance, altitude, and vegetation type (Schowalter and Ganio 1999; Schowalter et al. 2003; Richardson et al. 2005; Richardson et al. 2010; Schowalter et al. 2014). In Puerto Rico, Collembola are well known in comparison to other groups of soil arthropods. However, not all Collembola species from LEF have been identified.

In a recent survey between 2014 and 2015, we identified 16 families (sensu Deharveng 2004; Soto-Adames et al. 2008; Bellinger et al. 1996–2019), 37 genera, and probably 60 species, of which 15 are new. As a result of this survey, the inventory of Collembola in the LEF increased to 44 genera and 70 species. The purpose of this paper is to describe a new species of *Furculanurida* Massoud, 1967, a genus not previously reported from Puerto Rico.

The genus *Furculanurida* was created to relocate *Micranuridaafricana* Massoud, 1963 because of the development of its furcula (Massoud 1967). The main characters of this genus have been discussed previously (Palacios-Vargas and Gao 2009; Queiroz and Fernandes 2011; Zon et al. 2014, Neves et al., 2019), and some species are dubiously placed within *Furculanurida* (Table 1). To date, the genus has 16 nominal species distributed in the Neotropical, Ethiopian, and Nearctic regions, with seven, six, and one described species, respectively. Reported in the Neotropical Region are *F.arawakensis* Thibaud & Massoud, 1983 from the Lesser Antilles; *F.guatemalensis* Palacios-Vargas & Gao, 2009 and *F.septemoculata* Palacios-Vargas & Gao, 2009 from Guatemala; *F.nessimiani* Fernandes & Mendonça 2002, *F.belemensis* Arlé & Rufino, 1976, *F.goeldiana* Arlé & Rufino, 1976, and *F.tropicalia* Queiroz & Fernandes, 2011 from Brazil; and *F.longisensillata* Najt, Thibaud & Weiner, 1990 from French Guiana.

**Table 1.** *Furculanurida* species with disputed generic placement and their taxonomic history.

**Species**	**Original genus**	**Generic placement** ^+^	**Character** ^++^
*africana* (Massoud, Z, 1963)	*Micranurida*	***Furculanurida*** (type species) Massoud 1967	Furcula developed
*arlei* Thibaud & Massoud, 1980	*Furculanurida*	***Stachorutes*** Weiner and Najt (1998)	Presence of a microsensillum on Ant IV, mandible with only 2 teeth and a reduced furcula with very small mucro
		*Furculanurida* Thibaud and Palacios–Vargas (2000)	Presence of Ant. IV with 8 thick sensilla on antennal segment IV and long sensorial setae on the body and a small tooth in the unguis
*ashrafi* (Yosii, 1966)	*Micranurida*	*Stachorutes* Deharveng and Lienhard (1983)	Furcula reduced
		*Furculanurida* Thibaud and Palacios–Vargas 2000	Presence of four teeth in the mandible
*furculata* (Salmon, 1956)	*Kenyura*	***Furculanurida*** Massoud (1967)	Furcula developed and post-antennal organ present
*perplexa* (Salmon, 1956)	*Hypanurida*	*Furculanurida* Massoud (1967)	Reduced furcula
		***Hypanurida*** Thibaud and Palacios–Vargas (2000)	Reduced furcula and 3–4 setae in dens

^+^ Generic placement according to cited authors (current genus in bold). ^++^ Character to justify the placement in their current genus.

Initially, the genus *Furculanurida* was established using the combination of the following characters: postantennal organ (PAO) present, eyes absent, maxilla styliform, and furcula present (Massoud 1967). According to Queiroz and Fernandes (2011) species of the genus have Ant IV with a trilobed apical bulb, dorsolateral microsensillum present or absent, 4–7 sensilla, and long ordinary setae; PAO circular or elliptical with 4–22 vesicles; eyes from zero to eight per side; mandible with 2–10 teeth; maxilla styliform with two fused lamellae; tenent hair on tibiotarsi acuminated; ventral tube with 3 or 4 setae on each side; tenaculum with 2 or 3 teeth on each ramus; furcula complete, with well-developed dens and mucro; dens with 5 or 6 setae on each side; mucro separated from dens and with two tapering lamellae, sensilla on body always long. However, the more recently described *Furculanurida* species include specimens without the full development of the furcula (Zon et al. 2014). Zon et al. concluded that *Furculanurida* can only be separated from *Pseudachorutes* Tullberg, 1871 by a conditional combination of characters, i.e., “eyes less than 8+8, or, when 8+8, microchaeta ms absent on Ant. IV”. Similarly, differences with *Stachorutes* Dallai, 1973 would be “mucrodens complete, or, if mucro absent, ms absent on Ant. IV” (Zon et al. 2014: 496). Therefore, these genera need an extensive revision to clarify the morphological differences between them.

## Materials and methods

### Abbreviations

**a.s.l** above sea level

**Abd** abdominal segment

**Ant** antennal segment

**Cx** coxa

**Fe** femur

**M** long macroseta

**ms** microsensillum

**mi** microseta

**PAO** Postantennal organ

**S** sensillum

**Sgd** dorsal guard sensillum of Ant. III

**Sgv** ventral guard sensillum of Ant. III

**ss** sensorial seta

**Th** thoracic segment

**Ti** tibiotarsus

**Tr** trochanter

**VT** Ventral Tube

The material used to describe the species was collected during the Collembola microhabitats project at the Luquillo Mountains, as part of a survey conducted in three forest types. Collembola were extracted using Berlese-Tullgren funnels into 95% ethanol. They were cleared using Nesbitt solution and fixed on slides using Mac André II solution (Mari Mutt 1979). The slides were then dried in a slide warmer at 45–50 °C for seven days. Finally, each specimen was labeled with its collecting data. Specimens were examined with a Leica DM500 phase-contrast microscope. The drawings were made with the aid of a drawing tube. All the type material is deposited at International Institute for Tropical Forestry laboratory.

## Taxonomy

### Furculanurida bistribus sp. nov.

Taxon classificationAnimaliaCollembolaNeanuridae

2E559586-AD58-504A-9859-7B515D086689

http://zoobank.org:act:FE803C73-165E-42D6-9588-1343C9984A61

Figures 1 , 2 , 3–8 , 9–13 , Tables 1 , 2

#### Type material.

**Holotype** (female on slide) and 8 **paratypes** (2 males, 4 females, and 2 juveniles, each one on slides). Puerto Rico, Luquillo, Luquillo Mountains, Pico del Este, 18°16’17”N; 65°45’40”W; 987.6 m a.s.l.; ex mosses, *Tabebuiarigida* forest type, from leaf litter and epiphytes, 04 Nov 2014, leg. CM Ospina.

#### Other material.

2 females on slides, Puerto Rico, Luquillo, Luquillo Mountains, Pico del Este, *Tabebuiarigida* forest type, epiphyte, 987.6 m a.s.l., 19 May 2015, leg. CM Ospina. 1 female on slide, Puerto Rico, Luquillo, Luquillo Mountains, Pico del Este, *Tabebuiarigida* forest type, epiphyte, 987.6 m a.s.l., 11 Feb 2015, leg. CM Ospina. 1 juvenile on slide Puerto Rico, Luquillo, Luquillo Mountains, Pico El Yunque, *Tabebuiarigida* forest type, leaf litter, 1044.8 m a.s.l., 4 Nov 2014, leg. CM Ospina. 1 female on slide Puerto Rico, Luquillo, Luquillo Mountains, Pico El Yunque, *Tabebuiarigida* forest type, leaf litter, 1044.8 m a.s.l., 19 May 2015, leg. CM Ospina.

#### Diagnosis.

Eyes 3+3 eyes. Post antennal organ in rosette with 5 or 6 vesicles. Ant IV with six sensilla. Seta a0 on head absent. Mandible with four teeth. Dens with three setae. Unguis without internal tooth.

#### Description.

**Figure 1.**
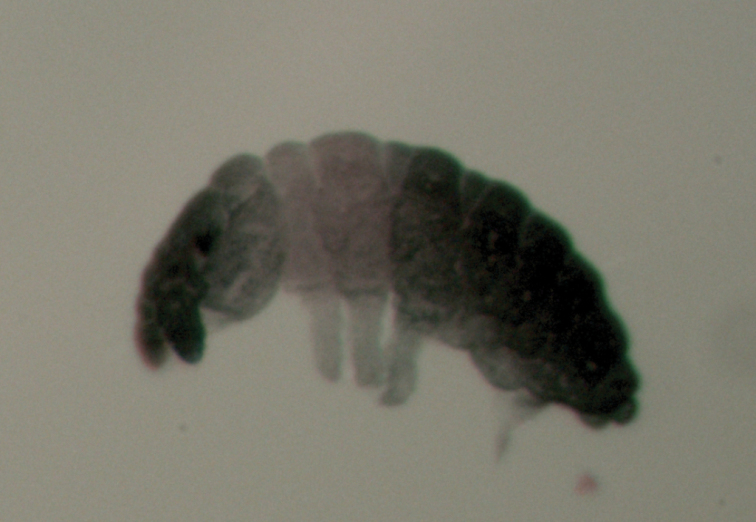
*Furculanuridabistribus* sp. nov. Specimen in ethanol 96%.

Average body length: adults 1009 µm (*n* = 5); juveniles 847 µm (*n* = 2). Specimens in ethanol with antenna and abdomen evenly grey, ocular patch dark; head, legs III, and furcula light grey; thorax, legs I and II white to light purple (Fig. 1). Granulation coarse. Body setae comprising short, smooth and thin setae, and long and smooth sensorial setae.

**Figure 2.**
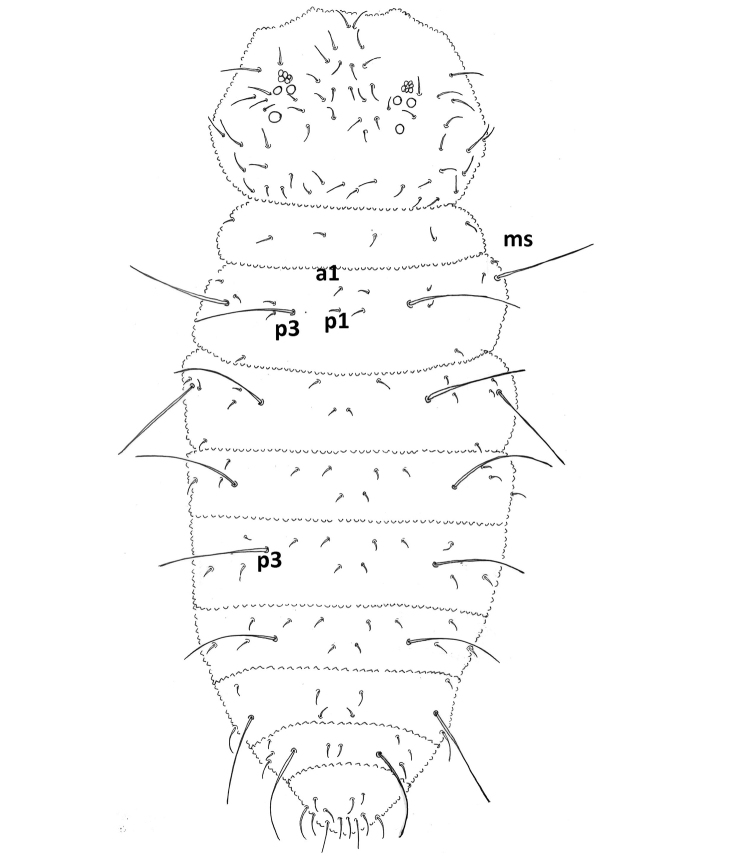
*Furculanuridabistribus* sp. nov. Dorsal chaetotaxy of body (female).

**Figures 3–8.**
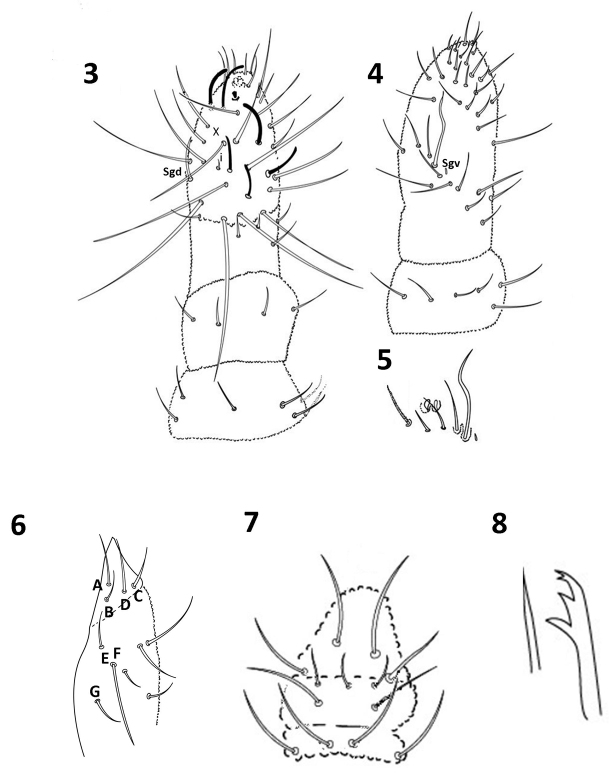
*Furculanuridabistribus* sp. nov. **3**Ant I–IV, dorsal view **4**Ant IV and III, ventral view **5** Sensorial Organ in Ant. III **6** Labium **7** Labrum **8** Maxilla and mandible.

Head: antenna shorter (0.6) than head diagonal. Ant III and IV fused dorsally, ventral separation clearly marked. Ant IV dorsally with trilobed apical vesicle, six subcylindrical thin sensilla and 14 long setae; subapical organite present; dorsoexternal microsensillum absent (Fig. 3); no sensorial field ventrally on Ant IV (Fig. 4); Ant III sense organ with two small internal slightly bent sensilla, two subcylindrical guard sensilla, Sgv larger than Sgd, ventral microsensillum present (Figs 3, 5); Sgd apically displaced, towards Ant IV, aligned to S2 and S3; Ant II with 10 setae; Ant I with seven setae (Fig. 3). Eyes 3+3 on a pigmented eyepatch; PAO with five or six vesicles disposed as a rosette (Fig. 2). Head dorsal chaetotaxy as in Figure 2; seta a0 absent; d row with 4 setae, sd row with three setae; setae Oc 1–3 present; c setae absent; p1–3 setae present. Buccal cone elongate, labium with complete chaetotaxy, A to G setae, C and D apically displaced (Fig. 6). Pre-labral/Labral chaetotaxy 4/2322 (Fig. 7). Mandible with four teeth, two apical short and subequal, one medial and one basal large and subequal; maxilla styliform with one lamella (Fig. 8).

Dorsal chaetotaxy: ordinary setae smooth, distributed as in Figure 2. Th I with 2+2 setae; Th II and III with one dorsolateral seta posteriorly displaced. Sensory setae (s) clearly differentiated, in position p3 and p6 in Th II and III and in position p3 in Abd I–V; S-chaetotaxy formula = 022/11111.

**Figures 9–13.**
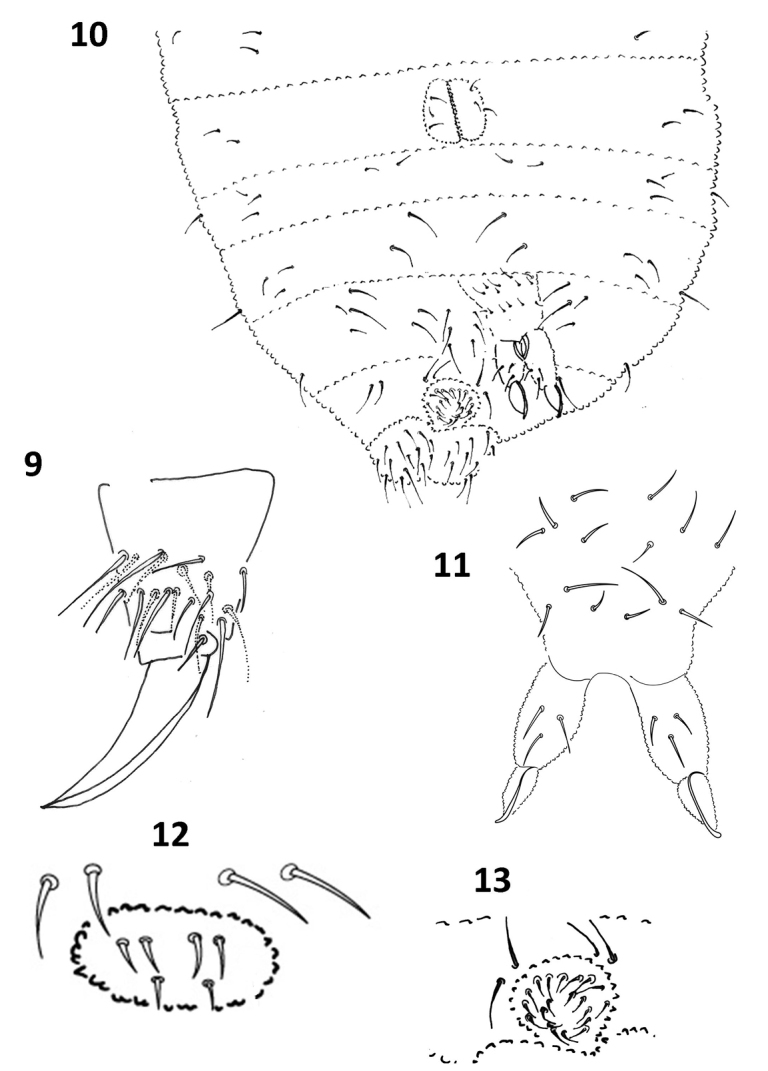
*Furculanuridabistribus* sp. nov. **9** Tibiotarsus II **10** Ventral quetotaxy **11** Manubrium and furcula **12** Female genital plate **13** Male genital plate.

Legs chaetotaxy: subcoxae 1, two; subcoxae 2, one; Cx, three; Tr, four; Fe, 10, and Ti 19 setae (Fig. 9). Seta M present between B4 and B5 without displacement. Tenent hair acuminate. Claws without teeth, unguis larger than Ti (ratio tibiotarsus: unguis = 1:1.2:); unguiculus absent.

Ventral chaetotaxy as in Figure 10. VT with 3+3 setae; tenaculum with 3+3 teeth and without setae; furcula well developed, manubrium with 14 setae, dens with three setae, mucro straight with a broad hook-like apex (Fig. 11). Ratio mucro: dens = 1: 1.3. Female genital plate with 2+2 pregenital setae, four circumgenital setae, and 1+1 eugenital setae (Fig. 12). Male genital plate with 2+2 pregenital setae, ten circumgenital setae, and 4+4 eugenital setae (Fig. 13).

#### Etymology.

*Bistribus*, Latin for two times three, in reference to the presence of 3+3 eyes and 3+3 setae on dens, diagnostic characters of the species.

#### Distribution.

This species is only the *Furculanurida* known from the Luquillo Mountains in the *Tabebuiarigida* forest type, on Pico del Este 18°16’17”N; 65°45’40”W; 987.6 m a.s.l. and Pico El Yunque 18°18’37”N; 65°47’26”W; 1044.8 m a.s.l.

#### Ecology.

*Furculanuridabistribus* sp. nov. was extracted from leaf litter and mosses in both dry and rainy seasons during November 2014, and May and August 2015.

### Identification key to the species of *Furculanurida* Massoud, 1967

**Table:** 

1	Eyes absent	**2**
–	Eyes present	**4**
2	Mucro developed, PAO with 8–10 vesicles	**3**
–	Mucro absent, PAO with 13–16 vesicles	***F.emucronata* Zon, Tano & Deharveng, 2014**
3	Internal tooth on unguis absent	***F.africana* Massoud, 1963**
–	Internal tooth on unguis present	***F.boiuna* Neves, Mendonça & Queiroz, 2019**
4	Eyes 8+8	***F.tropicalia* Queiroz & Fernandes, 2011**
–	Eyes 7+7 or less	**5**
5	Tenaculum with 2+2 teeth	**6**
–	Tenaculum with 3+3 teeth	**7**
6	PAO with 11 vesicles	***F.duodecimoculata* Thibaud & Massoud, 1980**
–	PAO with 6 vesicles	***F.nessimiani* Fernandes & Mendonça, 2002**
7	Setae on dens 3, internal tooth onunguis absent	***F.bistribus* sp. nov.**
–	Setae on dens 5 or 6, internal tooth on unguis present	**8**
8	Setae on dens 5	***F.langdoni* Bernard, 2007**
–	Setae on dens 6	**9**
9	Mandible with 10 teeth	***F.longisensillata* Najt, Thibaud & Weiner, 1990**
–	Mandible with 7 teeth or less	**10**
10	Ant IV with 7 sensilla, PAO with 4 vesicles, eyes 2+2	***F.furculata* Salmon, 1956**
–	Ant IV with 6 sensilla, PAO with more than 4 vesicles	**11**
11	Eyes 4+4 or less, mandible with 7 teeth	***F.arawakensis* Thibaud & Massoud, 1983**
–	Eyes 5+5 or 7+7, mandible with less than 7 teeth	**12**
12	Eyes 7+7	**13**
–	Eyes 5+5	**14**
13	PAO with 7-10 vesicles, mandible with 4 teeth	***F.goeldiana* Arlé & Rufino, 1976**
–	PAO with 15 vesicles, mandible with 2 teeth	***F.septemoculata* Palacios-Vargas & Gao, 2009**
14	PAO with 8 or 9 vesicles in a circular form	***F.belemensis* Arlé & Rufino, 1976**
–	PAO with 9 or more vesicles in an elliptical form	15
15	Setae on ventral tube 3+3, PAO with 9 or 10 vesicles	***F.grandcolasorum* Weiner & Najt, 1998**
–	Setae on ventral tube 4+4, PAO with 15 vesicles	***F.guatemalensis* Palacios-Vargas & Gao, 2009**

## Discussion

*Furculanuridabistribus* sp. nov. is placed in *Furculanurida* because many of its characters are similar with those of the other species of that genus, and it matches the current genus diagnosis: apical bulb trilobed, long setae present on Ant IV, maxilla styliform, furcula fully developed, and ordinary setae on the body short but sensory setae long (Queiroz and Fernandes 2011). Although the presence of one tooth on the unguis is observed in most *Furculanurida*, there are two exceptions where the unguis is toothless: *F.africana* and the new species described here. In any case, the inner tooth on the claw is usually considered as a specific, not a generic character (Zon et al. 2014). The antennal chaetotaxy is also useful to characterize the genus; the new species has antennal characters of *Furculanurida*: apical vesicle trilobed, microsensillum absent, and presence of six S-sensilla and long setae on Ant IV (Queiroz and Fernandes 2011). The low number of dental setae is a character shared with *Hypanuridaperplexa* Salmon, 1956, but the position of this species is controversial because of the reduction of the furcula (Queiroz and Fernandes 2011).

Although morphological characters, including furcal reduction, appear similar between some *Furculanurida* (including *F.bistribus* sp. nov.) and *Stachorutes* (Zon et al. 2014), the geographic separation of the two genera is remarkable. The genus *Furculanurida* was established for three sub-Saharan African neanurids: *Micranuridaafricana*, *Kenyurafurculata* Salmon,1956, and *Hypanuridaperplexa* (see Massoud 1967). Subsequently, other species were described or included in *Furculanurida* from the Lesser Antilles, Guatemala, Brazil, French Guiana, Tanzania, Morocco, Nepal, and Ivory Coast (Queiroz and Fernandes 2011; Zon et al. 2014). The genus, thus, conforms to a general Gondwanan distribution, with a few exceptions, like *Furculanuridalangdoni* which is found in North America. In contrast, *Stachorutes* exhibits mostly an Holarctic distribution, with species known from China, France, Poland, Russia, Slovakia, Spain, and North America, except for a single species from Africa (Tanzania) (Simon-Benito et al. 2005; Fanciulli et al. 2017).

According to Queiroz and Zeppelini (2017), there are key diagnostic characters that define two groups of Pseudachorutinae in the Neotropics, which are chaetotaxy of antennae, head, thorax, and tibiotarsi. *Furculanuridabistribus* sp. nov. exhibits many similarities with *Arlesia* group of genera. In the antennal chaetotaxy, the only difference was the absence of S10 *sensu*Queiroz and Zeppelini (2017). Regarding head and thorax chaetotaxy, the main characters are similar, i.e. the absence of setae c2 and c3 and the presence of p1, p2 and p3 on head, the presence of only 2+2 setae on Th I, and one posterior setae displaced laterally on Th II and III. In consequence, this description reinforces the need for a revision of *Furculanurida*, as not only *F.bistribus* sp. nov. but possibly other species of *Furculanurida* might present characters that fit the pattern displayed by the *Arlesia*-group of genera *sensu*Queiroz and Zeppelini (2017).

Despite the differences of the new species with the most recent genus diagnosis of *Furculanurida* (Queiroz and Fernandes 2011), we place *F.bistribus* sp. nov. in this genus because of the fully developed furcula in this species (Fig. 11) and its distribution, despite the number of setae on the dens. Inclusion of the new species in this genus thereby enlarges the generic diagnosis to include species with 3–6 setae on the dens. The characters that place the new species close to *Stachorutes* are of uncertain generic value and need more studies (Bernard 2007; Zon et al. 2014, Neves et al. 2019).

*Furculanuridabistribus* sp. nov. has this unique combination of characters: six sensilla on Ant IV, 3+3 eyes, three setae in the dens, and the absence of an internal tooth on the unguis, combined with its color pattern. Members of *Furculanurida* have between zero and eight eyes per side; *F.bistribus* sp. nov. has 3+3 eyes, though some specimens of *F.arawakensis* may have this number (usually 4+4 eyes). All the described species have a fully developed furcula, but more dental chaetae than the new species. Leaving aside the unique characters of *F.bistribus* sp. nov., it is more similar to *F.arawakensis* from which it differs by the presence of four teeth on mandibles (versus seven), less dental chaetae (3 versus 6), and the absence of tooth on unguis. The differences between all the species of the genus are summarized in Table 2.

**Table 2.** Main characters of the all known species of *Furculanurida*, including species moved to other genera.

**Species** ^+^	**Locality**	**Eyes**	**PAO vesicles**	**Ant IV sensilla**	**Ant IV ms**	**Md. teeth**	**Inner unguis tooth**	**Dens setae**	**Mucro**	**VT setae**	**Tenaculum teeth**
*africana*	Ivory Coast	0	8–10	6	-	9	absent	6	developed	-	-
*arawakensis*	Lesser Antilles	4(2–4)	5–9	6	absent	7	present	6	developed	3	–
*arlei* (*Stachorutes*)	Morocco	5		8	–	2	–	–	small	–	–
*ashrafi* (*Micranurida*)	Nepal	–	–	–	–	9	–	–	–	3	–
*belemensis*	Brazil	5	8–9	6	–	4–6	present	6	–	–	–
*boiuna*	Brazil	0	8–9	6	absent	7	present	5–6	developed	3	3
*duodecimoculata*	Morocco	–	11	–		4	–	–	–	3	–
*emucronata*	Ivory Coast	0	13–16	–	absent	–	present		absent	3	3
*furculata*	Rwanda	2	4	7		7	present	6	developed	–	
*goeldiana*	Brazil	7	7–10	7	–	4				–	
*grandcolasorum*	Tanzania	5	9–10		absent	3–6				3	
*guatemalensis*	Guatemala	5	15	6	present	4	present	6	developed	4	3
*langdoni*	USA	5	14–22	19	present	6	present	5	developed	4	3
*longisensillata*	French Guiana	6	6–7	6	absent	10–11	present	6	developed	3	–
*nessimiani*	Brazil			6	absent	4	–	–	–	3	–
*perplexa* (*Hypanurida*)	Rwanda	4	20–22		–	5	present	3–4	spiniform	–	–
*septemoculata*	Guatemala	7	15	6	present	2	present	6	developed	4	3
*tropicalia*	Brazil	8	8–10	6	absent	4	present	6	developed	3	3
*bistribus* sp. nov.	Puerto Rico	3	6	6	absent	4	absent	3	developed	3	3

^+^ The current genus is in parenthesis. – No information included in the original description.

## Supplementary Material

XML Treatment for Furculanurida bistribus
